# Myopia Control Efficacy and Long-Term Safety of a Novel Orthokeratology Lens (MESOK Study)—A Randomized Controlled Clinical Trial Combining Clinical and Tear Proteomics Data

**DOI:** 10.3390/jcm12093210

**Published:** 2023-04-29

**Authors:** Kai Yip Choi, Jimmy K. W. Cheung, Gigi T. K. Wong, Peter H. Li, Sonia S. H. Chan, Thomas C. Lam, Henry H. L. Chan

**Affiliations:** 1 Centre for Myopia Research, School of Optometry, The Hong Kong Polytechnic University, Hong Kong SAR, China; kaiyip.choi@polyu.edu.hk (K.Y.C.);; 2 Centre for Eye and Vision Research (CEVR), 17W Hong Kong Science Park, Hong Kong SAR, China; kawai.cheung@cevr.hk (J.K.W.C.);; 3 Research Centre for SHARP Vision (RCSV), The Hong Kong Polytechnic University, Hong Kong SAR, China

**Keywords:** myopia control, orthokeratology, contact lens, tear proteomics, children health

## Abstract

Myopia control efficacy and long-term safety of the Breath-O-Correct orthokeratology (OK) lens was evaluated in a 2-year randomized, single vision (SV) spectacle lens-controlled, single-blind clinical trial combining clinical and tear proteomics data. A total of 71 children (43 OK, 9.8 ± 1.3 years; 28 SV, 9.5 ± 1.4 years) completed the 2-year study. Axial length (AL), cycloplegic refraction, clinical safety parameters (best-corrected visual acuity, central cornea thickness, corneal endothelial health, ocular surface disease index), and quantitative tear proteomics were evaluated by masked examiners. Mean 2-year-normalized AL elongations in the OK and SV groups differed significantly (*p* = 0.03) and were 0.37 ± 0.37 mm and 0.60 ± 0.41 mm, respectively. OK-mediated myopia control efficacy was 37.1%. No significant difference was found in clinical safety parameters of both groups (*p* > 0.10), except for a thinner central corneal thickness in the OK group (*p* = 0.01). Proteomics revealed modest OK lens-mediated effects on immune response proteins, including an increased abundance of haptoglobin at 6 and 12 months and a decreased abundance of two proteins (neutrophil defensin 3 and histone 4) at 6 months. The changes were further validated using a high-resolution multiple-reaction monitoring (MRM^HR^) mass spectrometry. In summary, the Breath-O-Correct OK lens significantly reduced AL elongation in schoolchildren without adverse clinical effects or subclinical inflammatory responses.

## 1. Introduction

In the previous decades, the prevalence of myopia has drastically increased. It is estimated that approximately one-third of the global population suffered from myopia in 2020, and the prevalence is expected to increase to nearly half of the global population by 2050 [1]. Myopia prevalence is particularly high in East Asia, with over half of the population currently being myopic. Myopia, particularly high myopia, is a risk factor for various ocular diseases, including, but not limited to, macular degeneration, glaucoma, and retinal detachment, conditions typically leading to irreversible vision loss [2]. As well as permanent vision loss, the correctable myopia also brings an economic burden [3]. The loss of global gross domestic product from uncorrected refractive error was estimated at USD 202 billion in 2007 [4], which climbed to USD 244 billion in 2015 [5].

In view of the negative impact by the myopia epidemic, eye care practitioners have been striving to provide optimal myopia control regimens to school-age children with the aim of reducing myopia progression and preventing high myopia. Common strategies include low-dose atropine [6,7,8], spectacle lenses [9,10], contact lenses [11,12], and orthokeratology (OK) [13,14,15]. The efficacy of low-dose atropine, particularly at a 0.01% concentration, varies substantially between patients and may depend on pre-treatment myopia progression rates [8]. Among the various optical treatments, OK lenses are used overnight, when they reshape the cornea to a flatter curvature. Upon successful fitting and continuous wearing, users see clearly during the daytime without spectacles or contact lenses, making OK a popular myopia control intervention among physically active children [16].

OK is a safe myopia control intervention provided the lens care routine is adequately followed, i.e., adequate hand washing, careful lens insertion and removal, lens rubbing with cleanser, lens soaking in disinfectants, and regular protein removal. Otherwise, it may result in symptoms of pain, light sensitivity, and burning sensations. In a study that surveyed 86 randomly selected eyecare practitioners, clinical data from 1317 unique patients with 2202 lens orders and 2599 patient-years of lens wear were evaluated [17]. In such a large volume of dataset, only two cases of microbial keratitis were reported [17], indicating an incidence rate of 0.00077 over the patient-years. Despite the existing evidence, safety has been a significant concern for parents considering whether to adopt this efficacious myopia control regimen. In two cross-sectional surveys, over 75% of parents regarded potential injury as a major concern when considering OK treatment for their children, and overnight contact lens wear is perceived as risky by parents [16,18]. It is, therefore, important for eye care practitioners to have robust scientific evidence supporting the use of OK in children requiring myopia control interventions. In addition to clinical safety data, the scientific evidence may also include biomarker data evaluating subclinical inflammation.

Recently, we have reported the ocular surface status, tear biomarker response, and myopia correction effect of an OK lens with novel material and design [19], and the study was the first to demonstrate the feasibility of tear proteome characterization in clinical safety and efficacy studies. The results suggested that tear fluid proteome analysis with sequential window acquisition of all theoretical mass spectrometry (SWATH-MS) quantification can be used to assess the ocular surface condition. Notably, the abundance of several immune-related proteins, including proline-rich protein (PRR) 27 and immunoglobulin complexes, changed after 1 and 3 months of lens wear. The current follow-up study, which is a randomized clinical trial, investigated the long-term ocular clinical safety and myopia control efficacy of this OK lens in Chinese school-aged children relative to single-vision spectacle lens treatment. In addition, this study also monitored the tear proteome using SWATH-MS quantitation for an up to 1-year period after the start of lens wear.

## 2. Materials and Methods

### 2.1. Study Design

The current study adopted a single-blind, randomized, parallel-group, spectacle-controlled design to evaluate the myopia efficacy of Breath-O-Correct OK lens in Hong Kong Chinese schoolchildren (ClinicalTrials.gov Identifier: NCT03919396, first registered on 18 April 2019). The clinical trial information is available at https://clinicaltrials.gov/ct2/show/NCT03919396 accessed on 22 February 2023. The primary outcome was myopia control efficacy in terms of the annualized change in AL. The secondary outcome was long-term safety for OK wear, including both clinical measurements and lab-based proteomics measurements. All procedures followed the tenets of the Declaration of Helsinki and were approved by the Institutional Review Board of The Hong Kong Polytechnic University. This study also adhered to the guidelines of CONSORT.

### 2.2. Study Population

The participants were Hong Kong Chinese schoolchildren aged between 8 and 12 years. The inclusion criteria were spherical refractive error of −1.00 to −4.00 D, astigmatic error half of the spherical error, and the best-corrected visual acuity of LogMAR 0.10 or better in both eyes. The exclusion criteria were ocular abnormality (e.g., congenital cataract, strabismus), systemic diseases requiring long-term medications, history of refractive surgery, contra-indication for overnight OK lens wear, and history of OK lens wear or other myopia control strategies. Written consent and assent were obtained from the parents/guardians and the participants, respectively, before any study-specific procedures.

### 2.3. Randomization and Masking

After confirming eligibility, the participants were randomly allocated into the intervention group to receive OK treatment or the control group to receive SV spectacle treatment. The allocation ratio was set at 6:4 with an assumption that the dropout rate in the intervention group would be higher due to complex lens care procedures. The randomization process was conducted by one investigator (H.H.L.C.) using a computer-generated random sequence. During the follow-up visits, the experimenters performing efficacy and safety assessments were masked (G.T.K.W., P.H.L.).

### 2.4. Intervention

Participants in the intervention group received the Breath-O-Correct OK lens (SEED Co., Ltd., Tokyo, Japan). It is made of a novel material based on Toray’s original polymer technology, with methacrylate compound containing fluoride and silicone. The material is characterized by its high durability, elasticity, and flexural strength compared to other traditional rigid gas-permeable lenses (verified by JIS K7211-1 and ISO-18369-4). The material also offers high oxygen permeability that reaches 78 × 10^−9^ Dk/t. The lens profile was ready-made and designed with shallower sagittal depth and wider optical zone, which better suits Asian eyes. The participants in the SV group were prescribed with SV spectacle lenses (Stellify, Hoya Vision, Tokyo, Japan) with Super Hard Coat. Most participants were prescribed with a refractive index of 1.5, although 1.56 was chosen for refractive errors exceeding −4.00 D.

### 2.5. Sample Size and Power

In a previous 2-year randomized clinical trial evaluating OK treatment [13], the axial elongation were 0.36 ± 0.24 mm and 0.63 ± 0.26 mm for the treatment and control groups, respectively, equivalent to an effect size of 1.08. Assuming a more conservative effect size of 0.90, 5% error for both the Type I and Type II errors, and using an allocation ratio of 1.5 (6:4), the minimum number of participants in the OK and SV groups were 42 and 28, respectively. Based on prior experience, a dropout rate of 23% was assumed; hence, 91 participants were randomized overall.

### 2.6. Study Procedures

All clinical procedures were conducted in the Optometry Research Clinic of The Hong Kong Polytechnic University. A phone-interview and an on-site screening visit were arranged to evaluate the eligibility of the participants, followed by the randomization process. Baseline assessments included ocular biometry, cycloplegic refraction, ocular health assessments, and tear extraction using Schirmer’s strips for proteomic analysis. All participants were asked to return at 6-month intervals and were followed up for 24 months. The recruitment started in April 2019 and ended in April 2020. All follow-up visits were completed in April 2022. Because of COVID-19-related lockdown, some subjects could not attend the follow up visits as scheduled. However, the date of latest assessment of these subjects coincided with the subsequent visit schedule. Hence, these subjects would be classified as lost-to-follow-up in the former, but then re-joined in the latter.

Participants in the intervention group receiving OK had to return for extra visits for lens fitting, corneal topography, and ocular health assessments after the first overnight wear, and after 1 week, 2 weeks, 1 month, then every 3 months of lens wear. All first-overnight visits were scheduled within 2 h of lens removal in the morning such that topography and ocular health could be adequately evaluated. For other visits, the time was between 9am and 6pm, as the diurnal variation of AL would be negligible over the longitudinal study period. Here, 12 and 16 (time-matched) subjects from the OK and the SV lens groups, respectively, were randomly recruited for tear proteomics analysis at 6 months and 1 year.

### 2.7. Clinical Outcome Measures

AL was measured for at least 5 times by an optical biometer (IOLMaster 500, Carl Zeiss, Dublin, CA, USA) with signal-to-noise ratio > 2.0. Cycloplegic refraction was measured 30 min after the installation of 2 drops of 1% cyclopentolate, 5 min apart, using an open-field auto-refractor (NVision K5001, Shin-Nippon, Osaka, Japan), which was converted into SER by adding half of the cylindrical refractive error onto the spherical refractive error.

OK clinical safety measures included BCVA measured by a self-illuminating Sloan ETDRS chart (Precision Vision, Woodstock, IL, USA), CCT measured by Lenstar (Haag-Streir, Bern, Switzerland), corneal endothelial health (CD, CV, and CH) measured by CEM-530 (Nidek Co., Ltd., Gamagori, Aichi, Japan), and an OSDI questionnaire.

### 2.8. Tear Samples Collection

**Figure 1:**
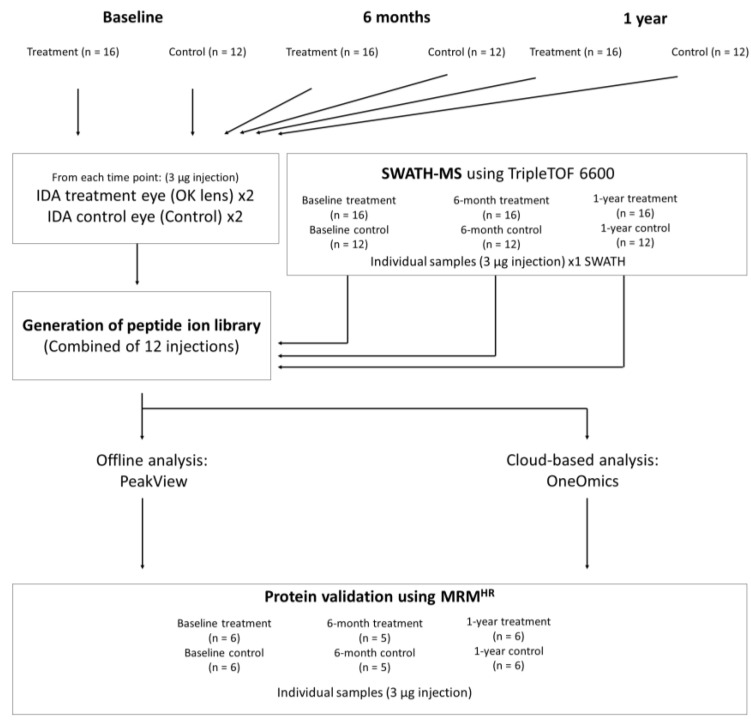
The experiment workflow of tear proteomics in this study. Tear samples were collected at baseline, 6 months, and 1 year (OK: n = 16 and SV: n = 12) using the Schirmer’s strip. Samples were prepared with the S-trap protocol for detecting differential protein expression using IDA and SWATH-MS. Acquired data were processed using (1) offline analysis (PeakView) and (2) online cloud-based analysis (OneOmics), and proteins with differential abundance between treatment groups were validated using a targeted-MRM^HR^ MS approach.

Tear samples were collected from subjects using a Schirmer’s strip placed at the temporal lower conjunctival sac of the subject without anesthesia. The subject’s eye was kept steady during the collection to minimize reflex tearing. At least 10 mm tears were collected, and strips were immediately dried with a heater and stored in a conical screw cap microcentrifuge tube at room temperature (RT) for storage. The overview of the experiment workflow is shown in Figure 1.

### 2.9. Tear Protein Extraction and Liquid Chromatography-Mass Spectrometry (LC-MS)/Mass Spectrometry (MS)

Tear sample preparation for MS and the LC-MS settings were described in our previous study [19,20]. In brief, the strip’s top end (beyond the 0 mm mark) was discarded, and the strip with tear fluid was cut into 1 mm interval spaces and placed into a new Eppendorf tube. A total of 100 μl of sodium dodecyl sulfate protein lysis buffer was added to the tube, and the mixture was incubated at 1000 rpm for 1 h at 25 °C in the thermomixer. Afterwards, the supernatant was transferred to a new tube and centrifuged for 30 s at RT.

Both IDA and data-independent analysis (DIA/SWATH-MS) were acquired with the TripleTOF^®^ 6600 quadrupole time-of-flight (QTOF) mass spectrometer (Sciex, Framingham, MA, USA) coupled to an Eksigent 415 nano-LC system (Sciex, Framingham, MA, USA). For IDA runs, equal amounts of peptides from each sample were pooled into the OK group and SV group within their respective time points and were loaded (3 μg load) onto a C18 trap column (PepMap100, 5 μm, 100 Å, 100 μm i.d. × 20 mm, Thermo Fisher Scientific, Waltham, MA, USA) at a flow rate of 2 μL/min for 15 min with duplicate runs. It was then separated with a C18 nano-LC column (5 µm, 100 µm i.d. × 300 mm, Column Scientific, Xiamen, China) at a flow rate of 350 μL/min running with a 3 h total gradient. For DIA/SWATH-MS, equal amounts of peptides (3 μg per sample, OK group: n = 16, SV group: n = 12) were loaded onto the MS with a setting of 100 variable isolation windows in a looped mode. It was set over the mass range of 100 to 1800 *m*/*z* with an accumulation time of 30 ms, resulting in a total duty cycle < 3 s.

### 2.10. Ion Library Generation and SWATH-MS Analysis

Twelve IDA injections were combined and searched against a Homo sapiens UniProt database (updated on 8 April 2021, 20,379 entries) using ProteinPilot (v5.0, Sciex, Framingham, MA, USA) for protein identification. Trypsin as the digestive enzyme, cysteine alkylation using iodoacetamide, “thorough” search effort, and biological modification were applied. Proteins and peptides identifications were filtered with a 1% FDR setting. This combined library was used as the ion library for SWATH-MS quantitation. For more comprehensive data analysis, apart from using the OneOmics platform in our recent study [19], an offline manual approach was also used to analyze data acquired from SWATH-MS, as follows:

(1) Offline analysis with PeakView (v2.2, Sciex, Framingham, MA, USA). The peptide spectral library was generated using the identified peptides from the peptide fragments’ peak extracted by SWATH Acquisition MicroApp 2.0 in Peakview. Up to 10 peptides per protein, 6 transitions per peptide, 90% peptide confidence threshold, 1% FDR, with a 10 min extracted ion chromatogram (XIC) extraction window and 75 ppm width settings, were selected for processing. Processed data were normalized using the most likely ratio (MLR) method and exported using MarkerView (v1.3, Sceix, Framingham, MA, USA) for protein fold change calculation. Proteins that passed the 1% FDR and with more than 1 quantifiable peptide were used for quantification. Volcano plots were drawn using VolcaNoseR using the R program [21].
(2) Cloud-based analysis with OneOmics. All the raw files (IDA and SWATH-MS) were uploaded to the OneOmics cloud-based analysis platform for protein library generation and spectral matching using PeakView. Up to 10 peptides per protein, 6 transitions per peptide, 90% peptide confidence threshold, 1% FDR, with a 10 min XIC extraction window and 75 ppm width settings, were selected for processing. Shared proteins and peptides with modifications were excluded from quantification, and the processed data were normalized using the MLR method. Proteins that passed the 1% FDR and with more than 1 quantifiable peptide were used for quantification.

### 2.11. Protein Confirmation Using MRM^HR^-MS

The transition list of targeted peptides and MRM^HR^ acquisition methods was created using Skyline (v20.2.0.286, MacCoss Lab, Seattle, WA, USA) [22]. MRM^HR^ acquisition was acquired using a QTOF mass spectrometer (Sciex, Framingham, MA, USA). Equal amounts (3 μg) of digested samples (BL: n = 6, 6 months: n = 5, 1 year: n = 6) were loaded onto a C18 nano trap column (PepMap100, 5 μm, 100 Å, 100 μm i.d. × 20 mm, Thermo Fisher Scientific) by loading buffer (0.1% Formic acid, 2% Acetonitrile in water) at 2 µL/min for 15 min. It was then separated on a C18 nano-LC column (100 µm × 30 cm, C18, Column Scientific, China) using an Ekisgent 415 nano-LC system at a flow rate of 350 nL/min with the following gradients: 0–0.25 min(s): 5%B, 0.25–45 min: 10%B, 45–60 min: 20%B, 45–60 min: 28%B, 60–65 min: 45%B, 65–75 min: 80%B, 75–90 min: 5%B. DIA mode was acquired with the mass range of 100 *m*/*z* to 1800 *m*/*z* scan. An accumulation time of 29 ms was set for each fragment ion, resulting in a total duty cycle of 3.0 s. Raw data of MRM^HR^ results were processed using MultiQuant (v3.0, Sciex, Framingham, MA, USA). The MQ4 algorithm was selected for automatic peak integration, resulting in a list of retention time, integrated peak area, peak height, and a signal-to-noise (S/N) ratio for each transition. The transition areas were then calculated from the average of 3 technical replicates (if possible, or else the top transition was selected). Transitions with a S/N ratio cut-off >20 were selected for calculation. Top transitions with a highest intensity area of ions were selected and averaged into each peptide, and the top peptides were then averaged for each protein and normalized with glyceraldehyde 3-phosphate dehydrogenase (GAPDH) for FC calculation in the 6 months lens wear group (FC: 0.89, *p* = 0.83, OK: 5; SV: 5, Welch’s *t*-test) and 1 year lens wear group (FC: 1.00, *p* > 0.99, OK: 6; SV: 6, Welch’s *t*-test). A full list of transitions and peptides used can be found in Supplemental Table S2.

### 2.12. Statistical Analysis

All statistical procedures for clinical data were performed using the SPSS 22.0 (IBM, Armonk, NY, USA). Owing to the strong correlation between right and left eyes (r = 0.89, *p* < 0.001), only clinical data from the right eye were analyzed. Furthermore, due to the COVID-19 lockdown, some scheduled follow-up visits were delayed, which was accounted for by evaluating the normalized change in AL using general linear model at 12 and 24 months, controlling for the age and baseline refractive error. Normalization was achieved by dividing the total AL elongation by the number of months between baseline and follow-up visit, then multiplying them by 12 and 24 for the interim and final analyses, respectively. The clinical safety parameters (BCVA, CCT, endothelial health, and OSDI) were compared at baseline and the final visit using mixed ANOVA. For proteomics analysis, proteins that have less than 1 peptide were filtered to reduce the chances of false-positive findings, with a fold change (FC) cut-off at ≥1.50 or ≤0.70 and a *p*-value ≤ 0.05 (Welch’s *t*-test), to be considered as differentially expressed using the offline analysis pipeline (PeakView), while an addition confidence level ≥ 75%, and repeatability ≥ 0.15 filters were also applied using the online clouded-based analysis platform (OneOmics).

## 3. Results

**Figure 2:**
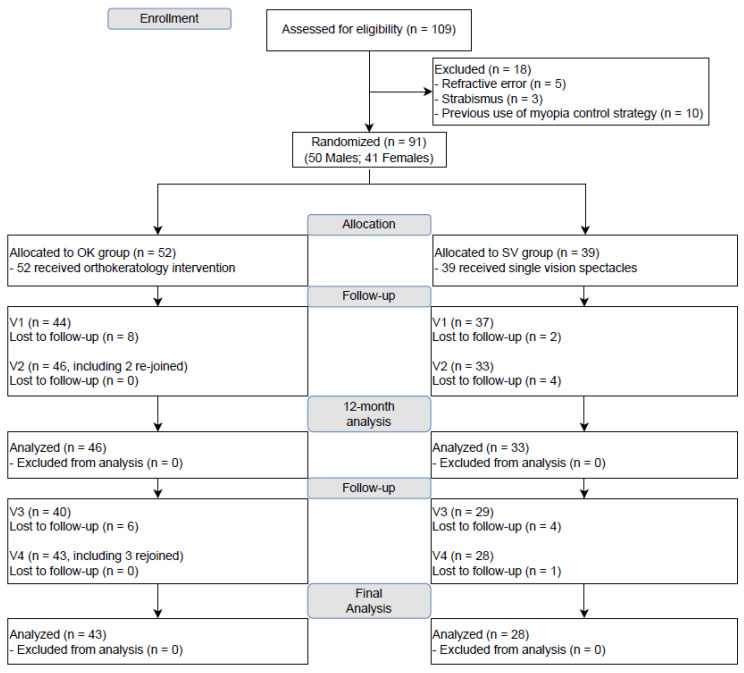
CONSORT diagram.

A total of 109 children were assessed for eligibility, and 91 were randomized to OK and single vision (SV) spectacles groups. Due to coronavirus disease 2019 (COVID-19)-related lockdown measures, some subjects had delayed visits relative to the protocol-defined visit window, and some were lost to follow-up. In total, 81 and 71 subjects completed the 12- and 24-month follow-up visits, respectively, and were included in the 12-month and 24-month efficacy analysis, respectively. The Consolidated Standards of Reporting Trials (CONSORT) diagram is summarized in Figure 2. Subjects lost to follow-up had similar baseline age, refractive error, and axial length (AL) as those included in the analyses (Table 1).

### 3.1. Changes in AL

**Figure 3:**
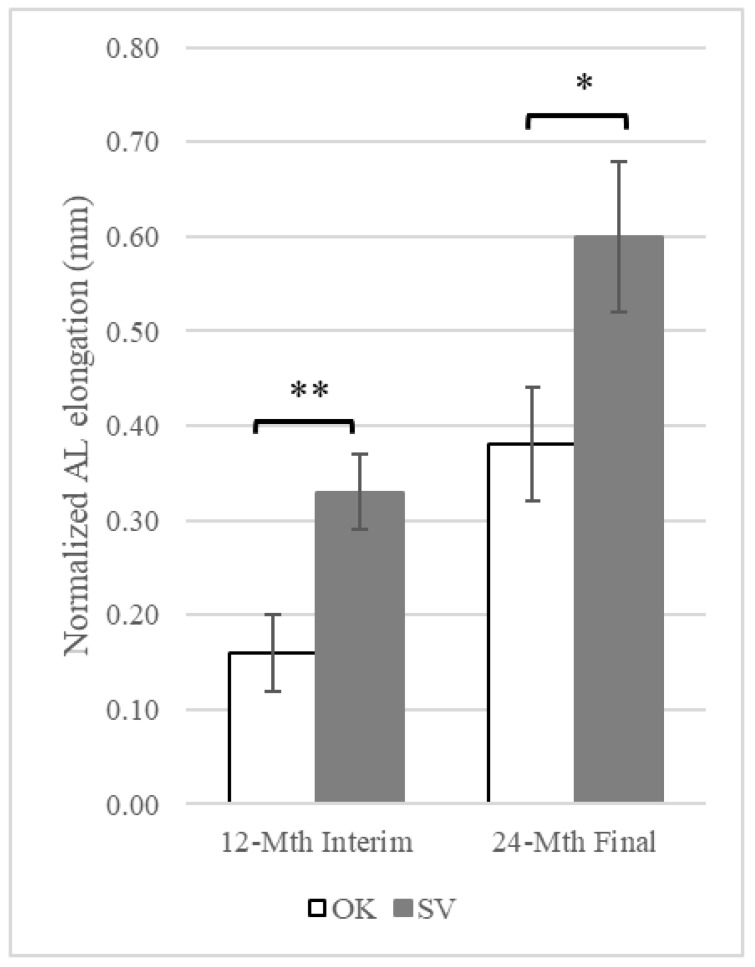
AL elongation in OK and SV groups. Error bars represent the stand error of means. Asterisk indicates *p* < 0.05; double asterisk indicates *p* < 0.01. AL: axial length.

At baseline, the refraction, in terms of spherical equivalence (SER) and AL, were similar between the OK and SV groups, as shown in Table 1. At 12 months, the mean 12-month normalized AL elongation was 0.16 ± 0.23 mm in the OK group and 0.33 ± 0.26 mm in the SV group. The between-group difference in 12-month normalized AL elongation was 0.17 mm after adjustment for covariates (F = 9.42, *p* = 0.003). In the final analysis, the mean 24-month normalized AL elongation was 0.37 ± 0.37 mm in the OK group and 0.60 ± 0.41 mm in the SV group, with a between-group difference of 0.22 mm after adjustment for covariates (F = 5.15, *p* = 0.03). The intervention provided 52.3% and 37.1% of myopia control efficacy at 12 and 24 months, respectively. Figure 3 demonstrates the normalized AL elongation in the OK and SV groups.

### 3.2. Long-Term Clinical Safety

Clinical safety parameters included best-corrected visual acuity (BCVA), central corneal thickness (CCT), corneal endothelial cell density (CD), cell variability (CV), cell hexagonality (CH), and ocular surface disease index (OSDI). As shown in Table 2, except for CCT, all clinical safety parameters were similar between treatment groups and remained stable over the study duration, without a significant main effect on time or a significant interaction effect between time and treatment groups. As expected, the OK group exhibited a significant central corneal thinning when compared with the SV group, with a significant interaction between time and treatment groups at both 12-month (*p* < 0.001) and final (*p* = 0.01) analyses.

### 3.3. Changes in Proteomics Profiling of Tear with 6-Month and 1-Year Lens Wear Using SWATH-MS

**Figure 4:**
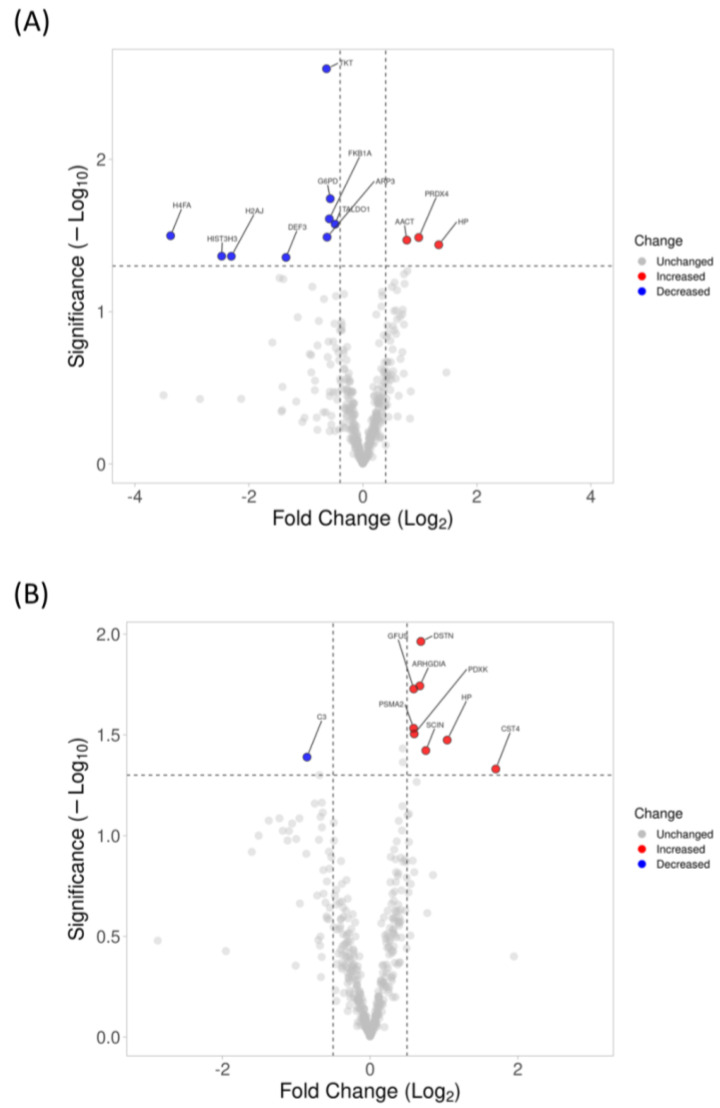
Volcano plots showing the statistical *p*-value with the magnitude of fold change between the OK group and SV group in (**A**) 6-month analysis, and (**B**) 1-year analysis using the offline PeakView analysis software. A total of 502 proteins were used for SWATH-MS quantification, and 12 and 9 proteins were found to be differentially expressed in 6-month and 1-year analyses, respectively (red: upregulated, blue: downregulated). Non-axial vertical dashed lines indicate a fold change of ±0.58 Log2 unit (±1.5-fold change), and the non-axial horizontal dashed line indicates a 1.30 −log10 *p*-value (*p* = 0.05), which is the significance threshold prior to logarithmic transformation.

A total of 581 unique proteins (6967 distinct peptides) at a 1% false discovery rate (FDR) were identified using information-dependent acquisition (IDA), and 502 common proteins were quantified across all the time points using SWATH-MS (OK: n = 16 and SV: n = 12). Using offline analysis with the Peakview software, at 6 months, three and nine proteins had increased and decreased in abundance, respectively, in the OK group relative to the SV group. At 12 months, eight and one proteins had increased and decreased abundance, respectively, in the OK group relative to the SV group (Figure 4). Only haptoglobin (HP) had increased abundance in the OK group relative to the SV group at both time points. The complete list of proteins with different abundances between treatment groups can be found in Supplemental Table S1. Only haptoglobin (HP) was found to be upregulated at both time points.

Using the OneOmics online cloud-based platform with the same criteria used in our previous study [19], no proteins had significantly different abundances between treatment groups at both time points due to the online algorithm for spectra alignment and dynamic retention time calibration as well as the more stringent filter applied (confidence level and repeatability threshold).

### 3.4. Protein Confirmation Using High-Resolution Multiple-Reaction Monitoring (MRM^HR^)

To validate differential abundance findings from SWATH-MS (after 6 and 12 months of OK lens wear), MRM proteomic analysis was performed for selected proteins in independent samples (6 months: n = 5, 1 year: n = 6). At 6 months, the fold change (FC; OK to SV group) in abundance of histone H2A.J was 0.06 in the MRM analysis, which was similar to the results of the SWATH analysis but did not reach statistical significance (*p* = 0.07). MRM analysis confirmed the SWATH findings of two other proteins, neutrophil defensin 3 (FC: 0.11, *p* = 0.02) and histone H4 (FC: 0.06, *p* = 0.05) at 6 months, with a significantly lower abundance in the OK group relative to the SV group (Table 3). At 12 months, only HP (FC: 1.51, *p* = 0.25) showed similar FC compared to SWATH-MS, but the findings were not significant in MRM^HR^ (a complete list of peptides and transitions used can be found in Supplemental Table S2).

## 4. Discussion

In this study, the Breath-O-Correct OK lens provided safe and efficacious myopia control in school-aged children. When compared with SV spectacles, treatment with the OK lens reduced AL elongation by 0.17 and 0.22 mm over 12 and 24 months, respectively. Generally, the treatment did not cause significant effects on ocular safety parameters, except for a reduction in central corneal thickness, which is expected based on the mode of action of OK lenses and has been as widely reported for other OK lenses [23]. Furthermore, the decreased abundance of DEF3 and H4, which are inflammation-related proteins, in the 6-month proteomic samples of the OK group, as the only significant findings that was consistent between two proteomic methods (SWATH and MRM), indicated adequate safety on the ocular surface upon long-term lens wear.

Myopia control efficacy of the OK lens was 52.3% over 1 year, and 37.1% over 2 years. This level of efficacy is comparable to efficacy reported in other OK studies and the efficacy of other myopia control strategies. In the Retardation of Myopia in Orthokeratology (ROMIO) study [13], the AL control efficacy was 55%, 32%, 29%, and 54% in the first, second, third, and fourth 6-month periods, for a mean efficacy of 43% over 2 years and a mean reduction in AL elongation of 0.27 ± 0.06 mm (0.36 ± 0.24 vs. 0.63 ± 0.26). In another randomized clinical trial investigating the effect of OK compression factor (OKIC) [14], the 2-year AL elongations were 0.35 ± 0.29 mm and 0.53 ± 0.29 mm for the increased compression group and the conventional group, respectively. Hence, the efficacy results of this study were within the efficacy of the ROMIO and OKIC studies. It is also worth noting that these comparisons warrant careful interpretation, as the studies may have differences in population, treatment protocol, and outcome measures.

The current study was conducted during the COVID-19 lockdown period. Multiple studies have demonstrated an increased myopia incidence and faster myopia progression in schoolchildren during the pandemic period [24,25], which was associated with decreased time outdoors and increased screentime [26]. Hence, effective myopia control became more critical during this period. Notably, studies evaluating the efficacy of low-dose atropine during the pandemic had mixed results; some studies found reduced efficacy relative to pre-pandemic studies [8,27,28], while other studies found efficacy consistent with prior studies [15].

Defocus-based myopia control interventions, however, had consistent efficacy before and during the pandemic, including defocus incorporated multiple segment (DIMS) lenses [10] and OK [15]. In this study, the difference in axial elongation was more prominent in the first than the subsequent 12 months. Potential reasons for this difference include COVID-19-related effects, and the higher dropout rate in the SV group (11/39, 28.2%) relative to the OK group (9/52, 17.3%).The main reason for dropout in the SV group was rapid myopia progression, as reported by the dropped-out parents, who then sought myopia control interventions other than SV spectacles. This violated our previous anticipation that higher dropout rate would be observed in the OK group because of the complex lens care procedures, and, hence, the subject allocation ratio (6:4) was a limitation in the study. This underestimation of myopia progression in the SV group is inevitable in OK studies with unblinded participants and may lead to an underestimation of the OK-mediated myopia control efficacy.

For a comprehensive proteomic analysis, the online cloud-based (OneOmics) analysis platform with stringent filters for peak alignments, confidence, and significate levels was used [19]. In addition, an offline analysis pipeline combining PeakView and MarkerView (Sciex, Framingham, MA, USA) was used in this study and in prior studies [29,30,31]. Proteins with differential abundance between treatment groups underwent validation testing using a target-MRM^HR^ approach to further reduce the chances of false positive results from the discovery stage. 

For a comprehensive proteomic analysis, the online cloud-based (OneOmics, Sciex, Framingham, MA, USA) analysis platform with stringent filters for peak alignments, confidence, and significate levels was used [19]. In addition, an offline analysis pipeline combining PeakView and MarkerView (Sciex, Framingham, MA, USA) was used in this study and prior studies [29,30,31]. Proteins with differential abundance between treatment groups underwent validation testing using a target-MRM^HR^ approach to further reduce the chances of false positive results from the discovery stage.

Using the offline analysis, the overall number of proteins with differential abundance between treatment groups was slightly higher relative to our previous study with shorter time points (1 month and 3 months) [19]. Apart from using different analysis platforms, this study had a larger sample size and analyzed samples from individual subjects in contrast to pooled samples in our previous study. The larger sample size and the use of individual samples may have contributed to the higher number of proteins with differential abundance. 

Two proteins in this study, neutrophil defensin 3 (DEF3) and histone H4 (H4), were found to have decreased protein levels in the OK group in the SWATH-MS analysis, a finding that was also confirmed using MRM^HR^ analysis in 6-month samples.

DEF3 is an antimicrobial peptide with a role in innate immunity against microbial pathogens [32]. During an immune stimulus, such as a bacterial or viral infection, neutrophils can be activated by cytokines and chemokines as a result of the infection, leading to an increased level of DEF3 [33]. The primary roles of DEF3 include direct membrane disruption [34,35,36] as well as the inhibition of the synthesis of the bacterial cell wall [37,38], causing the cell to leak or burst, hence, inhibiting the growth and killing bacteria. In ocular research, an increase in protein abundance of DEF3 in patients’ tears has been found under conditions, such as pterygium [39], ocular rosacea [40], and dry eye [41], which often include inflammation of the eye.

Histone H4 is a protein of the chromatin structure within the nucleus of cells and will be resealed as a damage-associated molecular pattern (DAMP) molecule when cells are damaged or undergo stress [42]. DAMPs can then activate the immune system and trigger an inflammatory response against infectious pathogens via the binding to pattern recognition receptors (PRRs) on phagocytes and inducing the production of pro-inflammatory cytokines and chemokines [43]. In conjunction with this, histone is also a major component of neutrophil extracellular traps (NETs) which are released from activated neutrophils during an infection outbreak. During the formation of NETs, nuclear materials are usually dissembled and intracellular organelle membranes are disintegrated, resulting in a cell-destructive process for the elimination of pathogens and microorganisms [44,45].

The finding of a decrease in DEF3 and H4 protein abundance in the current study in the OK group (6 months) suggested an absence of an inflammatory or immune response found after the introduction of the OK lens, indicating safe lens wear. The list of proteins with differential abundance in the SWATH-MS analysis (Table S1) can be used as a benchmark in future proteomic studies in patients with OK interventions.

## 5. Conclusions

In summary, the Breath-O-Correct OK lens effectively reduced AL elongation when compared with the SV lens control over 2 years, without adverse effects on clinical safety parameters or tear proteomics.

## Figures and Tables

**Table 1:** Baseline characteristics, represented by mean ± standard deviation.

All	OK	SV		
Subjects (n)	52	39		
Age (years)	9.8 ± 1.3	9.5 ± 1.4		
SER (D)	−2.67 ± 1.08	−2.50 ± 1.02		
AL (mm)	24.62 ± 0.94	24.42 ± 0.69		
	**Analyzed**		**Excluded**	
**12-month**	**OK**	**SV**	**OK**	**SV**
Subjects (n)	46	33	6	6
Age (years)	9.8 ± 1.3	9.3 ± 1.4	9.8 ± 1.3	10.3 ± 1.6
SER (D)	−2.59 ± 1.10	−2.50 ± 1.05	−3.37 ± 0.67	−2.47 ± 0.88
AL (mm)	24.57 ± 0.96	24.43 ± 0.73	25.08 ± 0.57	24.33 ± 0.49
**24-month**	**OK**	**SV**	**OK**	**SV**
Subjects (n)	43	28	9	11
Age (years)	9.8 ± 1.3	9.5 ± 1.4	9.9 ± 1.4	9.5 ± 1.6
SER (D)	−2.61 ± 1.07	−2.51 ± 1.03	−2.92 ± 1.18	−2.47 ± 1.03
AL (mm)	24.53 ± 0.93	24.52 ± 0.73	25.04 ± 0.87	24.17 ± 0.52

Baseline parameters did not differ significantly between treatment type or between subjects included and excluded from the efficacy analyses (unpaired *t*-test, all *p* > 0.05). OK: Orthokeratology; SV: Single vision; SER: spherical equivalence refraction; D: diopters.

**Table 2:** Clinical safety parameters.

	Baseline		12 Months		Interaction [F(*p*)]
	OK (n = 46)	SV (n = 33)	OK	SV	
BCVA	−0.02 ± 0.05	0.00 ± 0.07	0.04 ± 0.09	0.01 ±0.05	2.41 (0.13)
CCT *	550.3 ± 33.7	540.0 ± 34.0	531.2 ± 38.0	537.6 ± 34.7	15.84 (<0.001)
CD	3067 ± 232	3096 ± 159	3042 ± 224	2992 ± 200	2.26 (0.14)
CV	24.17 ± 3.10	23.61 ± 3.60	24.67 ± 3.85	25.89 ± 3.45	2.65 (0.11)
CH	68.13 ± 5.34	69.5 ± 5.15	68.53 ± 3.82	68.04 ± 5.00	2.08 (0.16)
OSDI	5.87 ± 4.71	8.41 ± 6.82	4.28 ± 4.28	5.56 ± 4.81	0.77 (0.39)
	**Baseline**		**Final (24 months)**		**Interaction [F(*p*)]**
	**OK (n = 43)**	**SV (n = 28)**	**OK**	**SV**	
BCVA	−0.01 ± 0.04	−0.01 ± 0.07	0.03 ± 0.10	0.00 ± 0.03	2.38 (0.13)
CCT *	547.5 ± 35.2	540.8 ± 36.4	538.5 ± 41.0	545.0 ± 36.0	8.56 (0.01)
CD	3051 ± 239	3100 ± 168	2973 ± 253	3005 ± 200	0.13 (0.72)
CV	24.10 ± 3.00	23.71 ± 3.88	24.48 ± 4.36	23.88 ± 2.72	0.05 (0.82)
CH	68.03 ± 5.27	69.50 ± 5.44	69.90 ± 5.21	68.48 ± 4.41	2.85 (0.10)
OSDI	5.80 ± 4.74	7.29 ± 5.01	4.74 ± 4.81	5.57 ± 5.76	0.21 (0.65)

Asterisk indicates a significant interaction between time and treatment groups in mixed ANOVA. BCVA: best-corrected visual acuity; CCT: central corneal thickness; CD: corneal endothelial cell density; CV: cell variability; CH: cell hexagonality; OSDI: ocular surface disease index.

**Table 3:** Two proteins with between-group differences in abundance in SWATH-MS and MRM^HR^ analyses at 6 months.

Protein Accession	Protein Name	Gene Name	Peptide Sequence Used for MRM^HR^	Transitions Used for MRM^HR^	MRM^HR^ (5 OK; 5 SV)		SWATH-MS (16 OK; 12 SV)	
					OK/SV FC	*p*	OK/SV FC	*p*
P59666	Neutrophil defensin 3	DEFA3, DEF3	IPAC[CAM]IAGER YGTC[CAM]IYQGR	+2y6, +2y7, +2y8 +2y4, +2y5, +2y6	0.11	0.019	0.39	0.044
P62805	Histone H4	H4C1, H4/A, H4FA, HIST1H4A	VFLENVIR	+2y6	0.06	0.051	0.10	0.032

FC: fold change.

## Data Availability

The data presented in this study are available on request from the corresponding author.

## Supplementary Materials

The following supporting information can be downloaded at: https://www.mdpi.com/article/10.3390/jcm12093210/s1, Supplemental Table S1: Proteins with differential abundances between treatment groups as found using SWATH-MS after 6-month and 1-year lens wear; Supplemental Table S2: Peptides and transitions of GAPDH and proteins with differential abundance in MRM^HR^ validation studies (A) 6-month lens wear (B) 1-year lens wear.
